# A contrast-enhanced ultrasound-based nomogram for the prediction of therapeutic efficiency of anti-PD-1 plus anti-VEGF agents in advanced hepatocellular carcinoma patients

**DOI:** 10.3389/fimmu.2023.1229560

**Published:** 2023-07-27

**Authors:** Chao Sun, Qian Wang, Lu Hou, Rui Zhang, Yu Chen, Lijuan Niu

**Affiliations:** ^1^ Department of Ultrasound, National Cancer Center/National Clinical Research Center for Cancer/Cancer Hospital, Chinese Academy of Medical Sciences and Peking Union Medical College, Beijing, China; ^2^ Department of Radiotherapy, National Cancer Center/National Clinical Research Center for Cancer/Cancer Hospital, Chinese Academy of Medical Sciences and Peking Union Medical College, Beijing, China

**Keywords:** hepatocellular carcinoma, programmed death receptor-1, contrast-enhanced ultrasound, nomogram, anti-VEGF (vascular endothelial growth factor)

## Abstract

**Background:**

There is no study focusing on noninvasive predictors for the efficacy of sintilimab (anti-PD-1) plus IBI305 (a bevacizumab biosimilar) treatment in advanced hepatocellular carcinoma (HCC).

**Method:**

A total of 33 patients with advanced HCC were prospectively enrolled and received sintilimab plus IBI305 treatment from November 2018 to October 2019. Baseline characteristics including clinical data, laboratory data, and tumor features based on pretreatment CT/MR were collected. Meanwhile, pretreatment contrast-enhanced ultrasound (CEUS) for target tumor was performed and quantitative parameters were derived from time–intensity curves (TICs). A nomogram was developed based on the variables identified by the univariable and multivariable logistic regression analysis. The discrimination, calibration, and clinical utility of the nomogram were evaluated.

**Results:**

Tumor embolus and grad ratio were significant variables related to the efficacy of sintilimab plus IBI305 strategy. The nomogram based on these two variables achieved an excellent predictive performance with an area under curve (AUC) of 0.909 (95% CI, 0.813–1). A bootstrapping for 500 repetitions was performed to validate this model and the AUC of the bootstrap model was 0.91 (95% CI, 0.8–0.98). The calibration curve and decision curve analysis (DCA) showed that the nomogram had a good consistency and clinical utility.

**Conclusions:**

This study has established and validated a nomogram by incorporating the quantitative parameters of pretreatment CEUS and baseline clinical characteristics to predict the anti-PD-1 plus anti-VEGF treatment efficacy in advanced HCC patients.

## Introduction

Hepatocellular carcinoma (HCC), with a 5-year survival rate of 5%–30%, ranks as the fourth most common malignant tumor and the second leading cause of cancer-related death (1, 2). The insidious onset and slow progression of symptoms usually result in delayed diagnosis of HCC. Considering the severity of HCC, only 10%–15% of HCC patients are eligible for surgical resection (3). In general, systemic treatment is the main option for advanced HCC patients (4). Anti-vascular endothelial growth factor (anti-VEGF) drugs were applied as standard systemic treatment agent, but the median overall survival (OS) ranges from 10.7 to 13.6 months (5–9).

Immune checkpoint inhibitors (ICIs), particularly antibodies targeting programmed cell death-1 (PD-1) or programmed cell death ligand-1 (PD-L1), have exhibited promising potential at improving tumor response and survival of HCC patients. The United States Food and Drug Administration (FDA) has approved anti-PD-1/PD-L1 for the treatment of advanced HCC (10). However, the efficacy of mono-immunotherapy remains limited. As first-line treatment, nivolumab monotherapy did not prolong the median OS compared with sorafenib monotherapy (11). The combination of ICIs and VEGF inhibitors is a promising strategy to fight tumors in a synergistic way. The VEGF inhibitor helps to induce the normalization of tumor vascularization, alleviate immunosuppression of tumor microenvironment, and increase the infiltration and activation of immune cells. Meanwhile, PD-1/PD-L1 inhibitors can enhance the stimulation of immune cells by targeting immune checkpoints (12–14). Recently, a series of clinical trials had demonstrated that ICIs plus anti-VEGF can result in more improvements in objective response rate (ORR), disease control rate (DCR), and progression-free survival (PFS) (15, 16). In 2020, FDA had approved the combined strategy as first-line treatment for advanced HCC.

In the background of precision medicine, it is urgent to identify the population who are likely to benefit from combined treatment. Imaging plays an important role in the management of HCC and has potential to provide noninvasive information for the prediction of treatment efficacy. Current studies mainly focused on applying imaging features to predict treatment response to mono-immunotherapy. Based on radiomics features extracted from pretreatment contrast-enhanced CT images, Yuan developed a nomogram to predict the anti-PD-1 treatment efficacy in patients with advanced HCC (17). Huang reported that the presence of the hyper-enhanced rim on the Kupffer phase images obtained from Sonazoid-contrast-enhanced ultrasound (Sonazoid-CEUS) is a promising biomarker to predict unfavorable response with anti-PD-1/PD-L1 therapy in HCC patients (18). At present, no noninvasive predictors for the efficacy of ICI plus anti-VEGF inhibitor treatment have been reported.

CEUS is a first-line modality in the management of HCC with high temporal resolution and high sensitivity to detect hypervascularization (19, 20). Different from contrast-enhanced CT/MR agents that deposit into extravascular space, ultrasound contrast agents are true intravascular contrast agents that are capable of quantification analysis of tumor perfusion information. Quantification parameters of CEUS had been widely used for the early evaluation or prediction of the response to antiangiogenic therapy in tumors with various types (21–24).

In order to screen population that might potentially benefit from combined treatments, our study develops a nomogram based on quantification parameters of pretreatment CEUS to predict the efficacy of ICI plus anti-VEGF inhibitor treatment.

## Materials and methods

This was a single-center prospective study approved by the ethics committee of the cancer hospital of the Chinese Academy of Medical Sciences (No.18-126/1704). All enrolled patients had given their informed consent.

### Patient selection and sample size estimation

From November 2018 to October 2019, 33 HCC patients treated with sintilimab (anti-PD-1) plus a bevacizumab biosimilar (anti-VEGF) were included in this study. The inclusion criteria were listed as follows: (1) patients who were aged ≥ 18 and diagnosed with HCC by histology or cytology; (2) with the presence of measurable lesions (≥1) proven by CEUS and contrast-enhanced CT/MR examination performed within 1 week before the start of combined treatment; (3) with a regular CT/MR follow-up duration ≥ 12 weeks; (4) patients in stage B or C according to the Barcelona Clinic Liver Cancer (BCLC) staging system; (5) with Eastern Cooperative Oncology Group (ECOG) performance status of 0 or 1; and (6) with Child–Pugh liver function scores ≤7. The exclusion criteria were as follows: (1) without baseline CEUS and CT/MR examination; (2) accepted locoregional therapy during follow-up; (3) currently has or had a history of malignant tumors besides HCC; (4) allergic to ultrasound/CT/MR contrast agents or other contraindication for ultrasound contrast agent application; and (5) incomplete follow-up.

The sample size estimation was based on the reported DCR in advanced HCC patients treated with anti-PD-1/PD-L1 plus anti-VEGF agents and on the principle of 10 outcome events per variable (25). According to a systematic review and meta-analysis, The DCR was 0.75 in PD-L1/PD-1 plus anti-VEGF agents. Using an estimated DCR of 0.75 in the study population and for two predictors (15), we aimed to enroll 27 HCC patients but actually enrolled 33.

### Dosage of anti-PD-1 plus anti-VEGF agents

Sintilimab was given intravenously at a fixed dose of 200 mg every 3 weeks and the bevacizumab biosimilar (IBI305) was given intravenously at a fixed dose of 7.5 mg/kg or 15 mg/kg. The incidence and severity of AEs were graded and recorded according to the Common Terminology Criteria for AEs version 5.0 (CTCAE 5.0).

### Clinical data and assessments of response to therapy

Baseline clinical characteristics including basic data, laboratory data, and abdominal CT/MR data of enrolled patients were collected and documented. Basic data included age, gender, BCLC stage, ECOG performance, and Child–Pugh score. Laboratory data included alpha-fetoprotein (AFP), total bilirubin (TBIL), alanine aminotransferase (ALT), aspartate aminotransferase (AST), and prothrombin time (PT). Target tumor size, tumor number, vascular invasion, and extra-hepatic metastasis status were documented according to baseline abdominal CT and/or MRI performed within 2 weeks before the initial treatment. Then, the follow-up abdominal CT was performed every 4 weeks after the initial treatment for treatment evaluation. Meanwhile, the baseline CEUS was performed within 1 week before the start of combined treatment and the details will be illustrated in *Examination procedure of CEUS*. Both CEUS and CT/MR chose the same HCC lesion as target lesion of each patient for evaluation. Based on anatomical location, a radiologist with over 20 years’ specialization in CEUS diagnosis was assigned to ensure that the target lesion observed by CEUS is consistent with the target lesion evaluated by CT/MR. If multiple HCC lesions exist in a patient, the biggest lesion that can be clearly revealed by CEUS was chosen as the target lesion.

Modified response evaluation criteria in solid tumor (mRECIST) was used to evaluate tumor response to treatment, and the classifications were listed as follows: (1) complete response (CR), disappearance of any arterial enhancement in target lesion; (2) partial response (PR), the total reduction of the diameters of the target lesions (arterial phase) by ≥30%; and (3) stable disease (SD), any cases that do not qualify for either PR or progressive disease (PD); PD, the diameter of the target lesion increased by at least 20% compared with the baseline value or the appearance of new lesions with enhancement in the arterial phase. Based on the response evaluation by two experienced radiologists (with 10 years’ experience in abdominal CT/MR diagnosis), the patients with CR, PR, or SD ≥ 12 weeks were classified as the non-PD group while the patients with PD during follow-up were categorized as the PD group.

### Examination procedure of CEUS

The conventional ultrasound and CEUS were performed using LOGIQ E9 (GE Healthcare, WI, USA) by an experienced radiologist (with 3 years’ experience in CEUS) within 1 week before the initial treatment. The frequency range of the probe was 3 to 5 MHz. The lyophilized powder of contrast agent Sono Vue (Bracco SpA, Milan, Italy) was reconstituted by adding 5 ml of 0.9% saline and shaking to form a homogeneous microbubble suspension. Before activating CEUS mode, conventional ultrasound was performed to screen the whole liver and choose target HCC lesions and best sonographic sections for observation. If multiple HCC lesions exist in a patient, the biggest lesion that can be clearly and completely presented by conventional ultrasound was selected as target lesion. The final sonographic sections for revealing target lesions were acquired by slightly adjusting on the basis of one of the standard sections introduced by the color atlas of ultrasound anatomy (26). The location (Couninaud liver segment), surrounding anatomic markers, and observing section of each target lesion were documented in order to facilitate the further identification in CT/MR images by a radiologist with over 20 years’ experience in HCC diagnosis. Then, a bolus of 2.4-ml suspension of the contrast agent was administered *via* antecubital vein. The CEUS mode and the chronograph were activated simultaneously. Continuous imaging was acquired immediately after injection of the contrast agent and lasted for 3 min. The imaging was presented as a dynamic video with a DICOM format. The dynamic videos were stored in LOGIQ E9 and backed up in a portable hard drive for further analysis.

### Quantification analysis of CEUS

The dynamic videos acquired from CEUS was analyzed using the built-in software of LOGIQ E9. Two radiologists (both with 3 years’ experience in CEUS) reviewed the dynamic video of each target lesion and selected the proper frame to draw the region of interest (ROI) including the tumor region (TR) and peritumoral region (PTR). The contour of each target lesion was manually drawn and the time–intensity curve (TIC) was automatically generated by the built-in software. Quantitative parameters generated from TIC included (1) time to peak (TtoPk), the time from zero intensity (right before the contrast arrives in the ROI) to maximum intensity; (2) peak intensity* (PI*), showing the difference between the peak intensity (PI) and baseline intensity (BI); (3) grad, the gradient from arrival intensity to PI, reflecting the average perfusion velocity; (4) area under curve (AUC), the area under TIC with the arrival intensity as baseline. The TIC obtained from TR (TIC-TR) and PTR (TIC-PTR) respectively generated corresponding quantitative parameters for each patient. The final variables used for binary logistic regression analysis was obtained by calculating the ratio of the parameters generated by TIC-TR to those generated by TIC-PTR.

### Development and internal validation of nomogram

Multivariable logistic regression was used to explore the relationship between variables and non-PD. Variables with *p*-value < 0.05 in univariate logistic regression were included in multivariable logistic regression. Redundant variable was excluded if collinearity existed. The variables with the variance inflation factors (VIFs) >5 indicated suspicious multicollinearities. Classification variables were set with a dummy variable. A nomogram model for predicting non-PD was developed using the independent risk factors identified by multivariable logistic regression analysis. The discriminatory ability of the model was evaluated using receiver operating characteristic (ROC) curve analysis. The predictive accuracy of the model was evaluated by a calibration curve. An internal bootstrap validation was performed using computer resampling for 500 repetitions of simple random sampling with replacement. Decision curve analysis (DCA) was performed to determine the clinical usefulness.

### Statistical analysis

R software (ver.1.4.1717, R Development Core Team) and SPSS 22.0 software (IBM Corporation, NY, USA) were used for statistical analysis. The *χ (* 2) test or Fisher’s exact test were used for the comparison of classification variables, whereas the independent-sample *t* test was used for the comparison of continuous variables. A *p*-value < 0.05 was considered statistically significant. SPSS was used for binary logistic regression analysis and R software was used to develop the predictive model and test the diagnostic performance of the model with the corresponding package.

## Results

### Baseline characteristics of patients in the PD group and non-PD group

A total of 33 patients were enrolled in this study from November 2018 to October 2019. Each enrolled patient received at least one cycle of sintilimab plus IBI 305 treatment. According to tumor response evaluation, the enrolled patients were divided into a PD group and a non-PD (PR+CR+SD) group as illustrated in the *Clinical data and assessments of response to therapy*. The baseline characteristics summarized from clinical data, laboratory data, and imaging data of baseline abdominal CT/MR are listed in **Table 1**. BCLC staging, ECOG performance status, and Child–Pugh liver function scores were not assigned as variables in baseline characteristics since these clinical data were taken as inclusion criteria. In addition, considering the IBI305 was given at two different doses of 7.5 mg/kg or 15 mg/kg, the dose of IBI305 was also listed as a variable in **Table 1**. Except for the variable of embolus, there were no significant differences in baseline characteristics including IBI305 dose between the PD group and non-PD group, indicating a good consistency between two groups.

**Table 1:** Baseline characteristics of patients in the PD group and non-PD group.

Characteristic	PD group (*n* = 11)	Non-PD group (*n* = 22)	*p*-value
Age at diagnosis (years)	59.09 ± 11.03	55.86 ± 11.97	0.46
Gender			
Male	9 (81.8%)	18 (81.8%)	0.671
Female	2 (18.2%)	4 (18.2%)	
AFP (ng/ml)			
<20	1 (9.1%)	5 (22.7%)	0.409
20–400	5 (45.5%)	7 (31.8%)	
>400	5 (45.5%)	10 (45.5%)	
Alb (g/L)	41.23 ± 7.69	44.21 ± 4.09	0.153
PLT (10^9^/L)	12.86 ± 0.98	12.30 ± 0.66	0.06
Tbil (μmol/L)	17.54 ± 9.79	13.94 ± 4.62	0.158
Tumor size (cm)	8.02 ± 5.86	8.40 ± 4.19	0.837
Tumor number			
<3	1 (9.1%)	8 (36.4%)	0.104
≥3	10 (90.9%)	14 (63.6%)	
Embolus			
Present	8 (72.7%)	4 (18.2%)	0.004
Absent	3 (27.3%)	18 (81.8%)	
Extra-hepatic metastasis			
Yes	10 (90.9%)	19 (86.4%)	0.593
No	1 (9.1%)	3 (13.6%)	
IBI305 dose			
7.5 mg/kg	7 (63.6%)	12 (54.5%)	0.453
15 mg/kg	4 (36.4%)	10 (45.5%)	

PD, progressive disease; IBI305, a bevacizumab biosimilar for anti-VEGF.

### Quantitative parameters generated based on baseline CEUS

**Figure 1:**
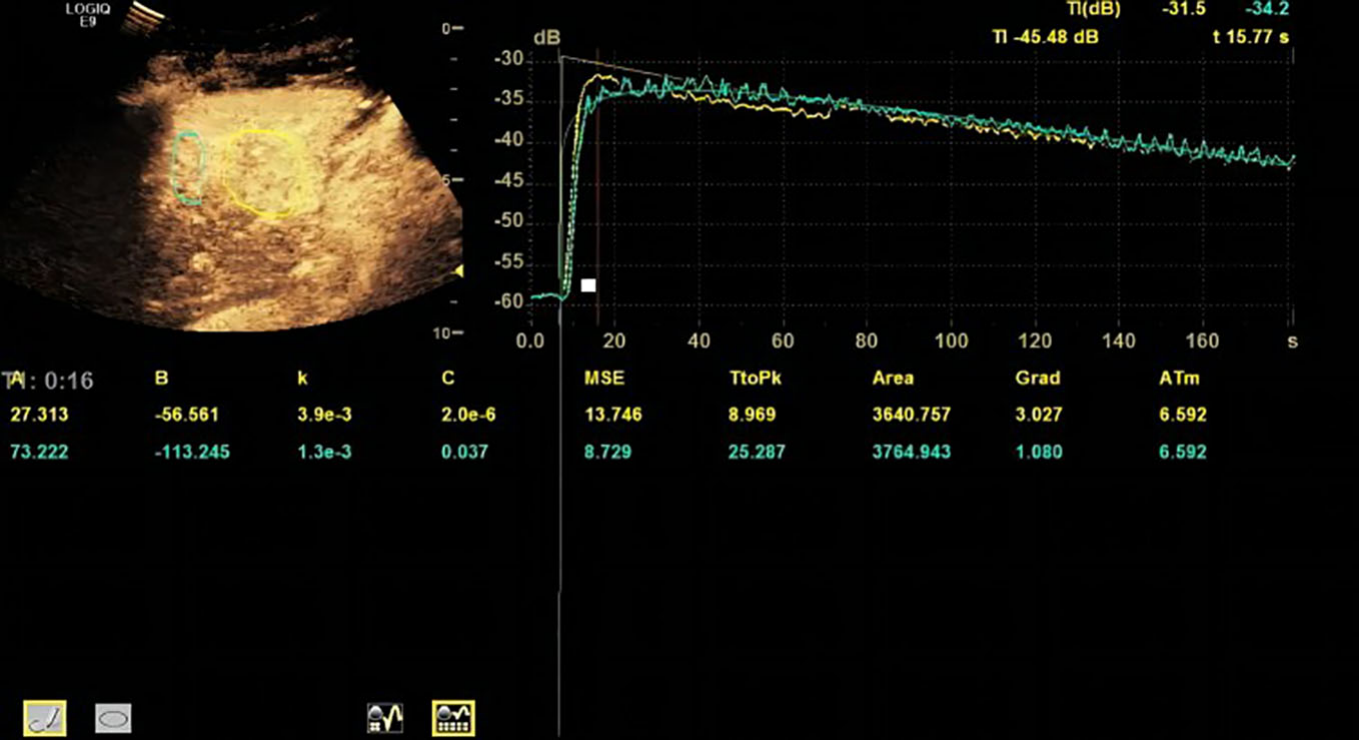
Representative image of contrast-enhanced ultrasound (CEUS) and time–intensity curve (TIC) of patients in the progressive disease (PD) group.

**Figure 2:**
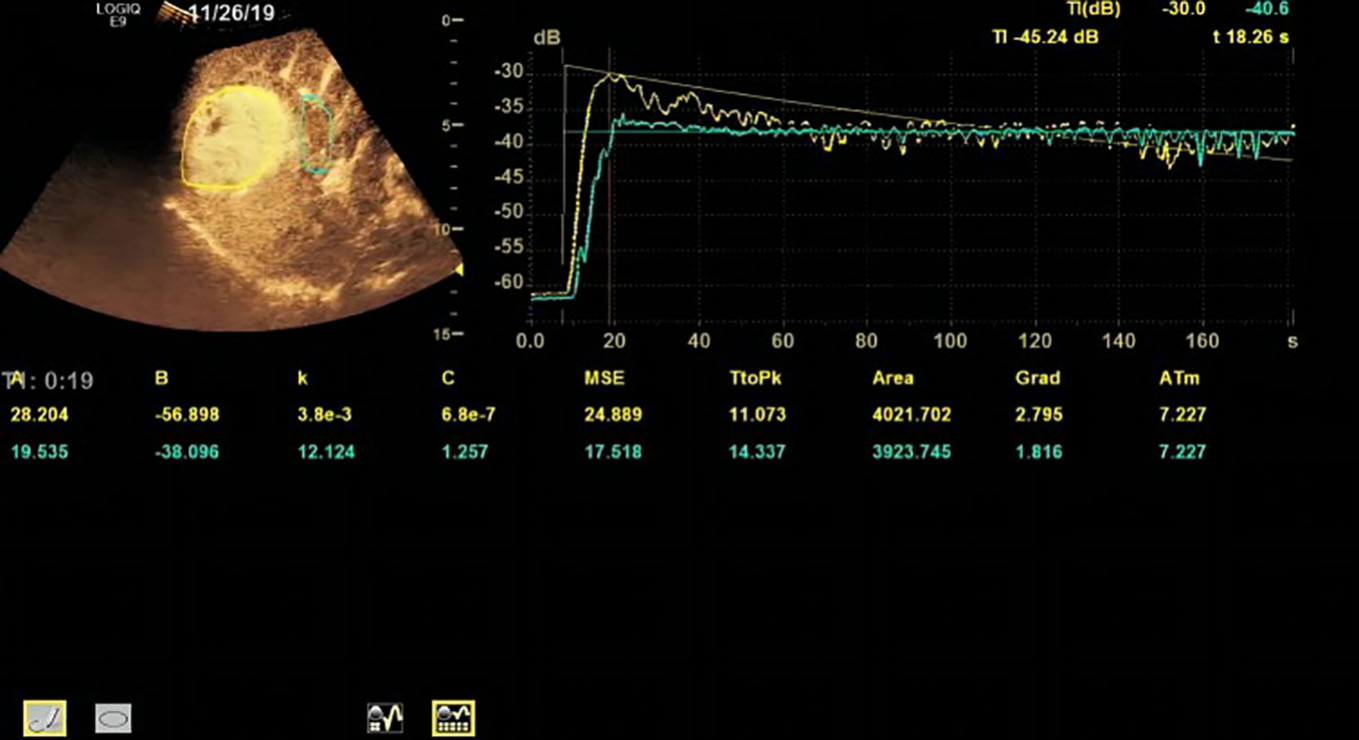
Representative images of CEUS and TIC of patients in the non-PD group.

Based on the TR (tumor region) and PTR (peritumor region), corresponding TICs were generated by built-in software. The TIC of TR was defined as TIC-TR while that of PTR was defined as TIC-PTR. Corresponding quantitative parameters of TIC-TR and TIC-PR are listed in **Table 2**. These quantitative parameters included TtoPK, PI*, grad, and AUC. The ratios of quantitative parameters of TIC-TR to those of TIC-PTR were calculated and are listed in **Table 2**. The representative images of CEUS and corresponding TICs are presented in **Figures 1**, **2**.

**Table 2:** Quantitative parameters based on TR/PTR in the PD group and non-PD group.

Parameters	PD group (*n* = 11)			non-PD group (*n* = 22)		
	TIC-TR	TIC-PTR	Related ratios	TIC-TR	TIC-PTR	Related ratios
TtoPK (s)	14.18 ± 3.21	29.32 ± 8.09	0.51 ± 0.15	17.32 ± 6.93	32.417 ± 10.05	0.54 ± 0.15
PI* (dB)	26.84 ± 5.22	23.98 ± 4.91	1.13 ± 0.22	23.94 ± 5.60	25.05 ± 5.30	0.97 ± 0.18
Grad	1.99 ± 0.69	0.87 ± 0.24	2.3 ± 0.62	1.46 ± 0.41	0.81 ± 0.31	1.8 ± 0.42
AUC	3,234.36 ± 732.45	3,117.00 ± 876.33	1.11 ± 0.50	2,887.81 ± 880.25	3,255.74 ± 846.78	0.90 ± 0.22

TIC, time–intensity curve; TR, tumor region; PTR, peritumor region; TtoPK, time to peak; PI, peak intensity; BI, baseline intensity; PI*, the difference between PI and BI; AUC, area under the operating characteristic curve. TIC-TR represents the TIC generated from TR; TIC-PTR represents the TIC generated from PTR; ratio represents the ratio of quantitative parameters of TIC-TR to those of TIC-PTR.

### Target tumor response to treatment

The response to treatment of target tumor was evaluated by the follow-up CT/MR. The follow-up duration of every enrolled patient was ≥12 weeks after initial treatment. While no CR was observed in enrolled patients, 11 patients experienced PR, 11 patients experienced SD, and 11 patients experienced PD. A DCR of 66.67% was obtained in our study (**Table 3**).

**Table 3:** Tumor response for enrolled patients after anti-PD-1 plus anti-VEGF treatment.

Tumor response	All patients (*n* = 33)
Complete response (CR)	0
Partial response (PR)	11 (33.33%)
Stable disease (SD)	11 (33.33%)
Progressive disease (PD)	11 (33.33%)
DCR (CR+PR+SD)	22 (66.67%)

### Nomogram for predicting non-PD

**Figure 3:**
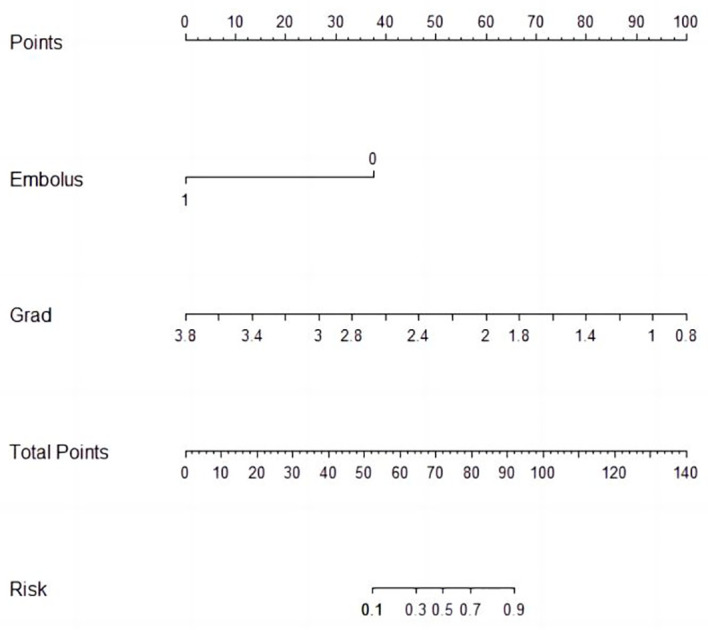
Nomogram prediction of non-PD.

**Figure 4:**
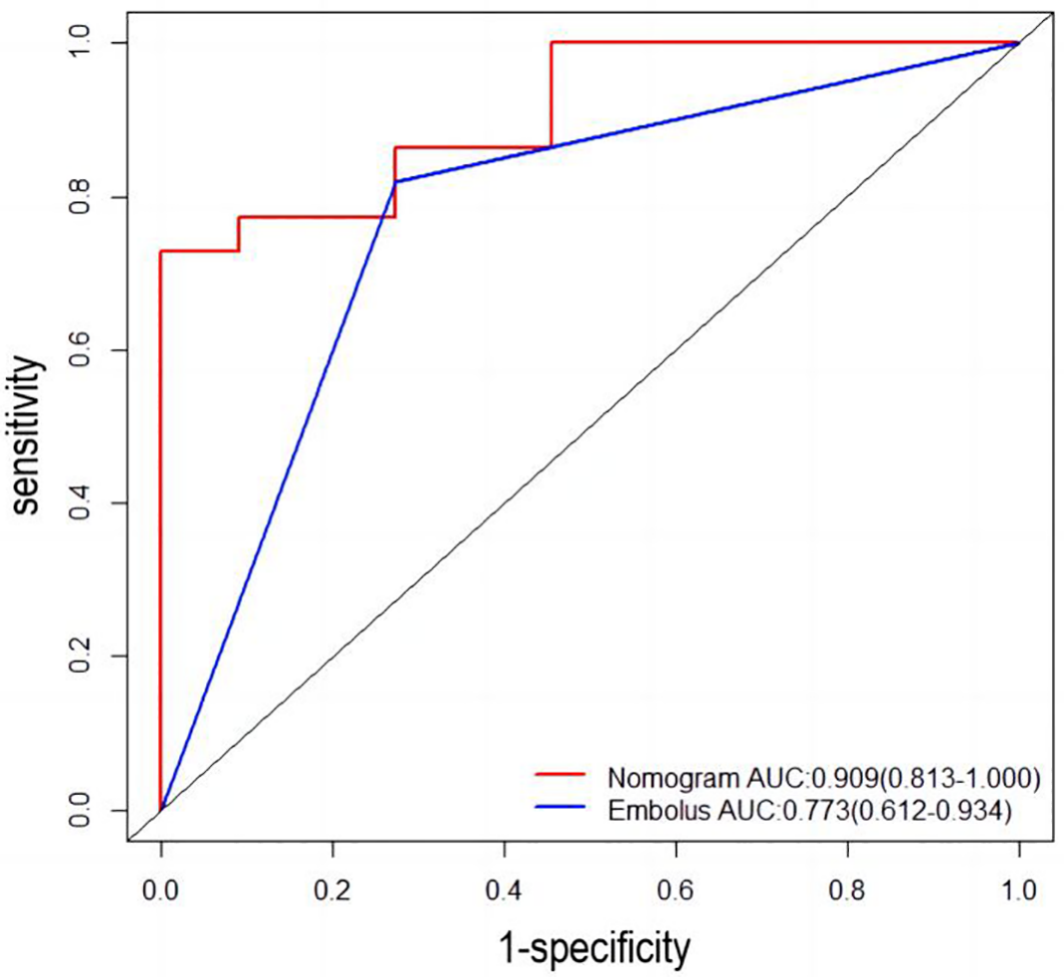
Receiver operating characteristic (ROC) curve. AUC: area under the receiver operating characteristic curve for the nomogram and for applying the embolus alone to predict non-PD.

Univariable and multivariable logistic regression analysis were performed to identify the independent variables related to non-PD (**Tables 4**, **5**). Finally, embolus and grad ratio were considered to be significant variables related to non-PD. The absence of embolus in portal vein and the lower value of grad ratio were predictive factors for non-PD. With these two variables, a nomogram was established and the probability of non-PD can be estimated (**Figure 3**). The discriminative ability was evaluated by ROC curve analysis. The area under the ROC curve (AUC) (AUC, 0.909 [95% confidence interval (CI), 0.813–1]) of the nomogram was higher than that for applying embolus alone (AUC: 0.773 [95% CI, 0.612–0.934]) (**Figure 4**).

**Table 4:** Univariable logistic regression analysis to identify risk factors for non-PD.

Variables	Odds ratio	95% confidence interval	*p*-value
Age	1.042	0.975–1.042	0.223
Gender (male)	1.00	0.153–6.531	1.00
Tumor size (cm)	1.227	0.995–1.513	0.055
Tumor number (≥3)	5.714	0.613–53.229	0.126
Embolus (present)	0.083	0.015–0.462	0.04
Extra-hepatic metastasis (yes)	0.708	0.145–17.218	0.708
IBI305 dose (15 mg/kg)	0.686	0.155–3.036	0.619
TtoPK ratio	5.431	0.03–978.72	0.523
PI* ratio	0.007	0.000–1.280	0.062
Grad ratio	0.136	0.023–0.789	0.026
AUC ratio	0.098	0.003–3.352	0.198

TtoPK ratio is defined as the ratio of the TtoPK obtained from TIC-PR to that obtained from TIC-PTR

PI* ratio is defined as the ratio of the PI*(PI-BI) obtained from TIC-PR to that obtained from TIC-PTR.

Grad ratio is defined as the ratio of the grad obtained from TIC-PR to that obtained from TIC-PTR.

AUC ratio is defined as the AUC obtained from TIC-PR to that obtained from TIC-PTR.

**Table 5:** Multivariate logistic regression analysis to identify risk factors for non-PD.

Variables	Odds ratio	95% confidence interval	*p*-value
Embolus (present)	0.015	0.001–0.338	0.008
Grad ratio	0.025	0.001–0.596	0.023

### Model validation

**Figure 5:**
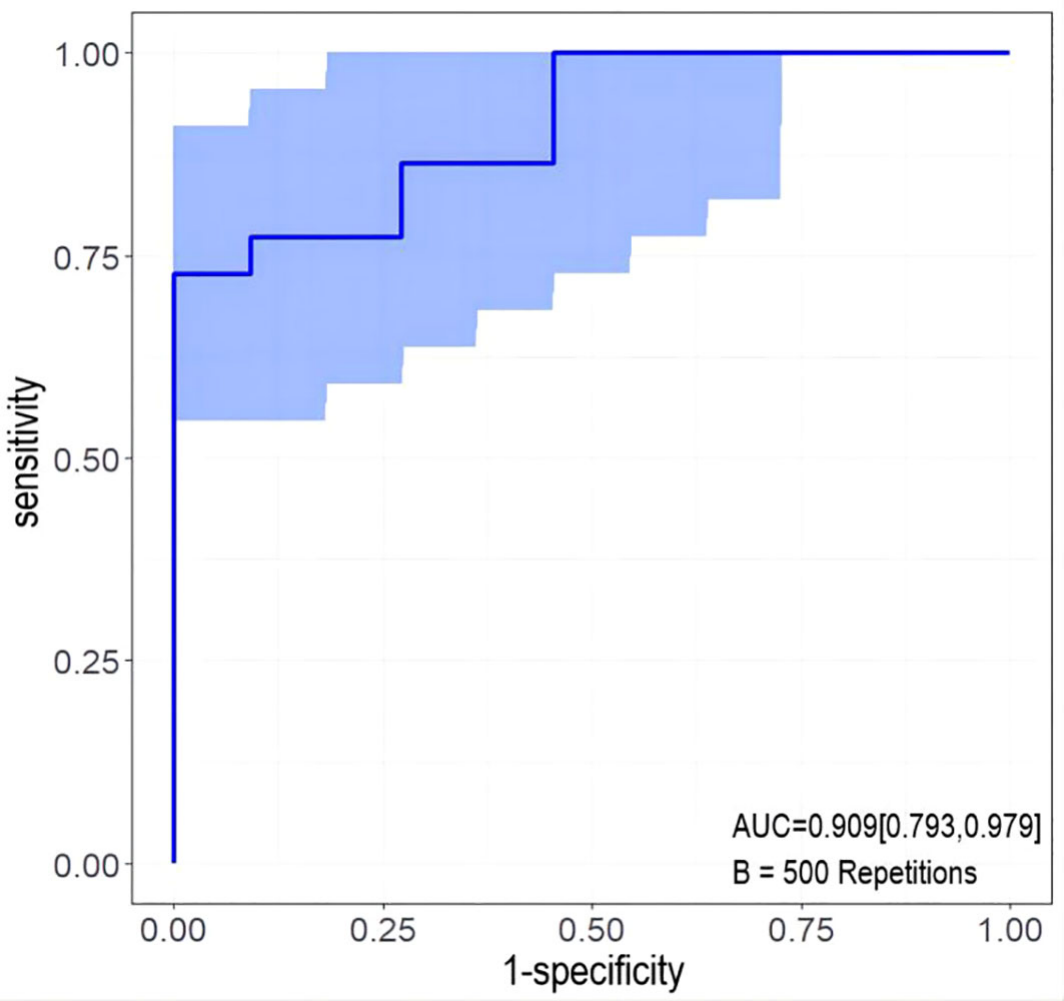
The ROC curve measured by bootstrapping for 500 repetitions and the AUC of the bootstrap stepwise nomogram. The snow blue area shows the 95% confidence interval of the ROC curve.

**Figure 6:**
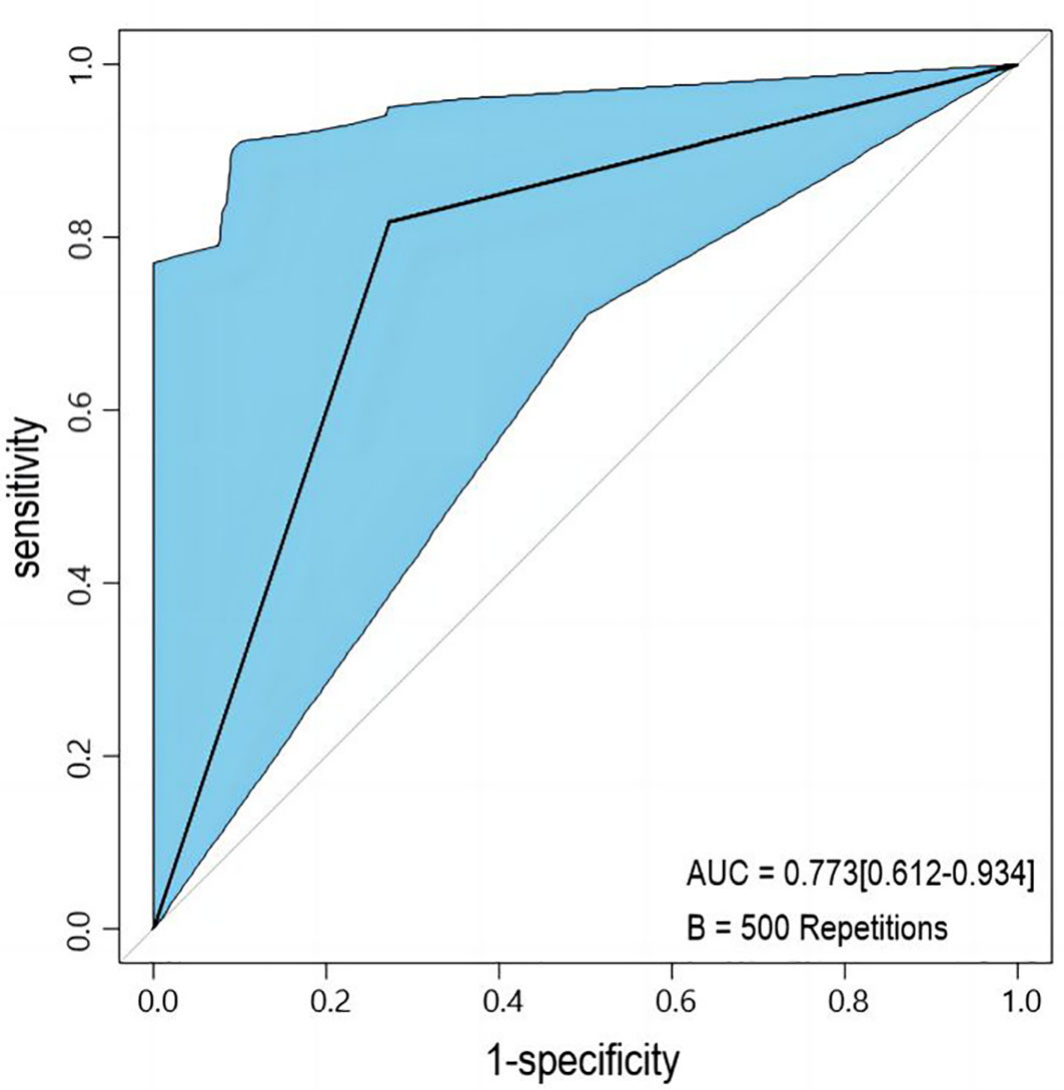
The ROC curve measured by bootstrapping for 500 repetitions and the AUC for applying embolus alone to predict non-PD. The lake blue area shows the 95% confidence interval of the ROC curve.

**Figure 7:**
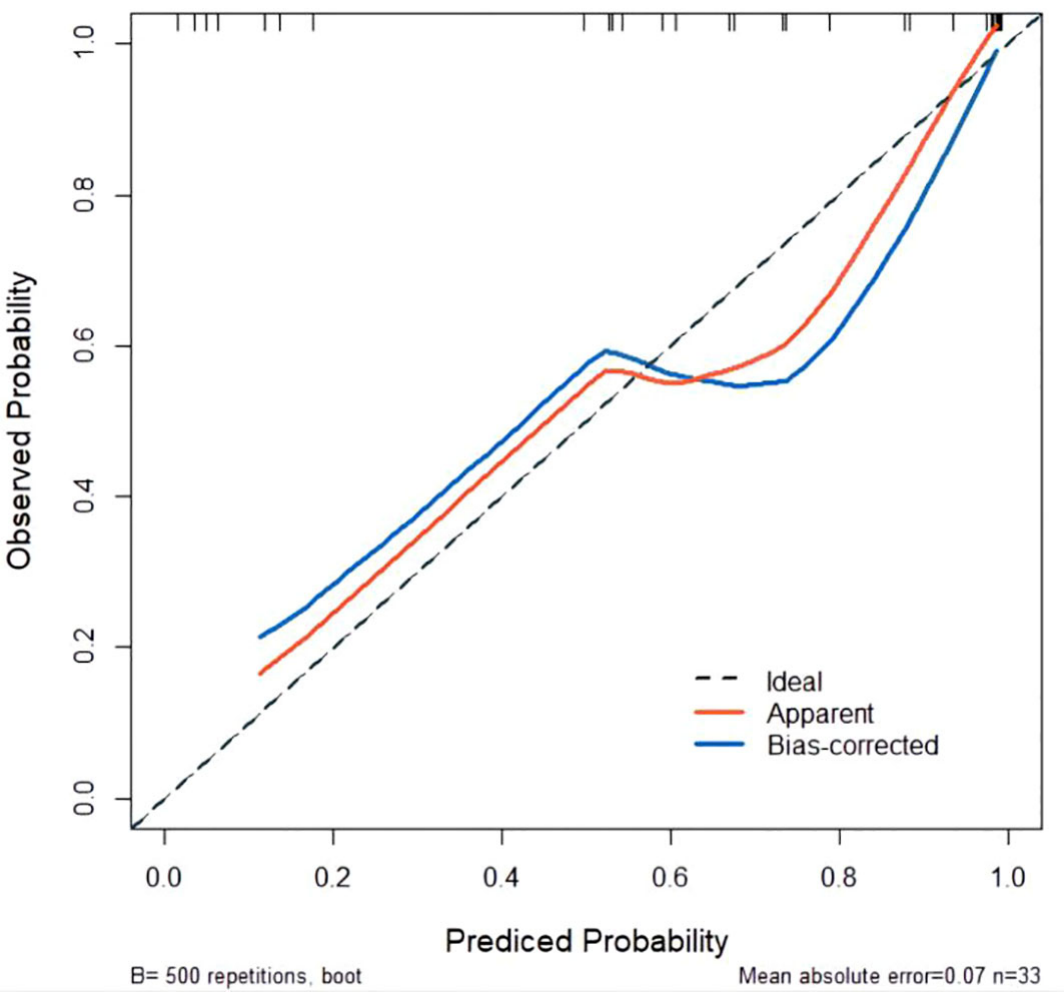
Calibration curve for predicted probability. The *X*-axis represents the probability of non-PD predicted by the nomogram. The *Y*-axis represents the actual probability of non-PD. The diagonal dashed line represents the ideal calibration line.

**Figure 8:**
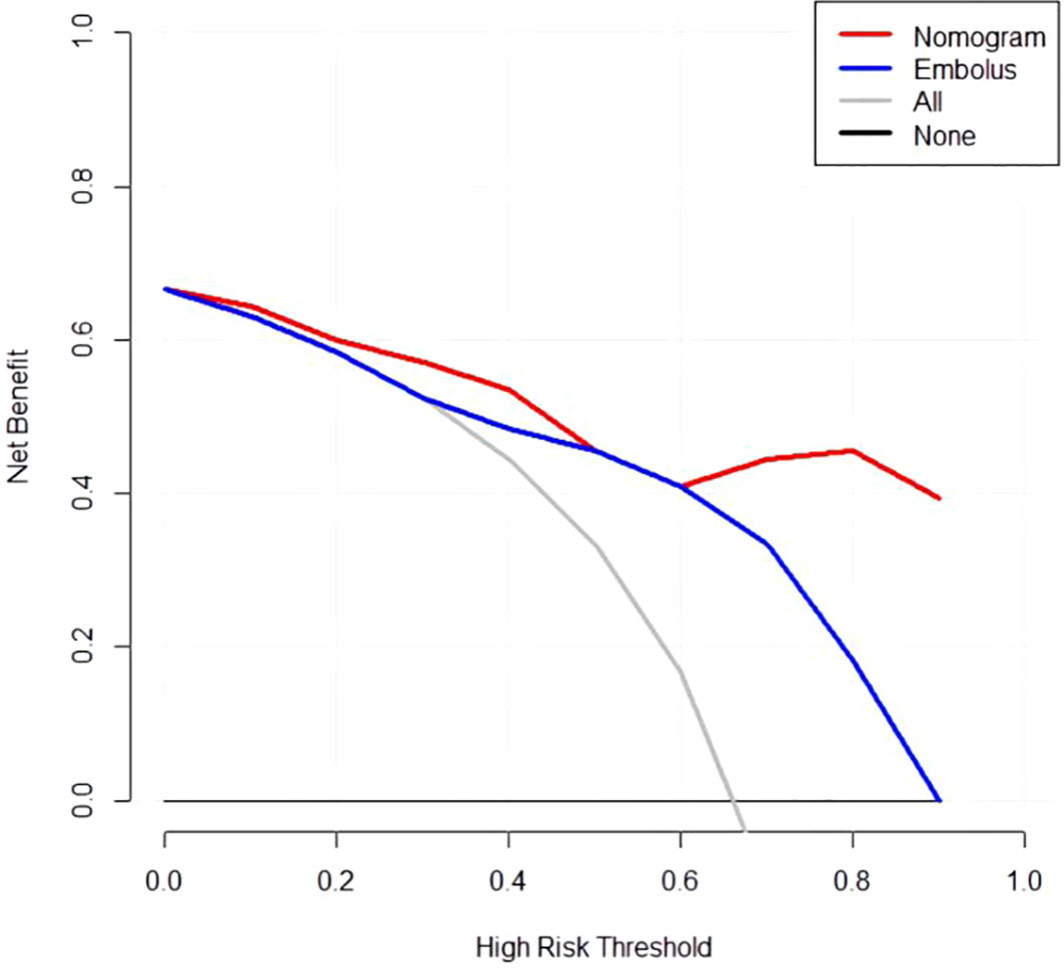
Decision curve analysis for the nomogram. Thin slash line: assume all patients are non-PD. Solid horizontal line: assume no patients are non-PD. The graph shows the expected net benefit per patient relative to the nomogram and embolus for the prediction of non-PD.

The developed nomogram was validated with internal bootstrap validation. The ROC curve was evaluated by bootstrapping for 500 repetitions and the AUC of the bootstrap nomogram was 0.909 (95% CI, 0.793–0.979) (**Figure 5**). Also based on the internal bootstrap validation, the AUC of the ROC curve for applying the embolus status alone to predict the therapeutic efficiency was only 0.773 (95% CI, 0.612–0.934) (**Figure 6**). Based on internal bootstrap validation, the calibration curve of the nomogram showed a good fitting with the idea curve. When the probability was less than 0.5, the nomogram may slightly underestimate the probability. When the probability was higher than 0.5, the nomogram may slightly overestimate the probability (**Figure 7**). The DCA showed a positive net benefit for the nomogram and embolus when a threshold probability was greater than 0.2. When compared with the net benefit achieved by applying embolus status alone to predict therapeutic efficiency, a better clinical utility was achieved after incorporating grad and embolus to establish the nomogram (**Figure 8**).

## Discussion

In this study, a CEUS quantitative parameter-based nomogram was developed and validated by bootstrap method to predict the anti-tumor efficacy in advanced HCC patients treated with sintilimab plus IBI305. By incorporating the variables of embolus and grad ratio, the nomogram achieved a good performance in predicting the probability of non-PD after anti-PD-1 plus anti-VEGF treatment.

Compared with anti-PD-1/PD-L1 monotherapy, the combined strategy of PD-1/PD-L1 inhibitors plus anti-VEGF agents achieved more clinical improvements for advanced HCC patients in ORR (0.26 vs. 0.21), DCR (0.75 vs. 0.59), and PFS (6.2 months vs. 4.19 months) according to the results of the meat analysis based on recent clinical trials (15). However, patients with unfavorable response to this combined strategy still existed. Studies focusing on exploring the biomarkers that aid in predicting the response to anti-PD-1 plus anti-VEGF agents are urgently needed. Generally, tumor neovascularization significantly differs from normal vasculature due to the presence of dilation, distortion, and formation of abnormal division branch, leading to corresponding blood perfusion patterns (27). The anti-VEGF agent can induce the normalization of tumor vascularization and thereby result in the changes of blood perfusion patterns (28, 29). Tumor-associated macrophages (TAMs) were considered to play an important role in immunotherapy resistance (30). As we know, TAMs can be divided into M1-like and M2-like subtypes. The high ratio of M1-like TAM to M2-like TAM can lead to a better long-term prognosis of cancer patients (31). The immune resistance can also be partially attributed to the predominant presence of M2-like TAM in the tumor environment (TME) (32). In patients with advanced HCC, high infiltration of M2 macrophage was considered to be associated with resistance to anti-PD-1 monotherapy (33). A research team from our institution recently reported that the tumor infiltration of M1 macrophages may serve as a potential predictive biomarker for anti-PD-1 plus anti-VEGF therapy in patients with advanced HCC (16). It is worth emphasizing that the M2-like TAMs is also associated with the microvessel density in tumor (31). This view was further supported by the finding that close association was observed between TAMs and tumor angiogenesis during cervical cancer progression (34). In well-differentiated HCC, tumor vascularity was also proved to be correlated with M2-like TAM count (35). Thus, the microvessel intensity in tumor is potentially useful to predict the immune resistance in TME by indicating the proportion of M2 TAMs. For now, no non-invasive predictors were mentioned for predicting the efficacy of the treatment using ICIs plus anti-VEGF agents in advanced HCC patients.

CEUS is a non-invasive imaging modality using a contrast agent consisting of gas bubbles that are small enough to transverse through pulmonary vasculature and finally reach the target organ vasculature. Different from CT/MR contrast agents, the ultrasound contrast agent is a true intravascular contrast agent without deposition into the extravascular space and has the potential to reflect the vascular distribution and intensity without the concerns of ionizing radiation. Zheng reported that a good correlation (*r* = 0.624, *p* < 0.001) was obtained between the quantitative CEUS variable (maximum intensity, namely, PI) and the intratumoral microvessel density (MVD) estimated based on surgical tissue sections stained with CD34 (36). By revealing the changes of microvessel perfusion, quantitative CEUS had been widely used in the early evaluation or prediction for the treatment efficacy of anti-VEGF monotherapy (24, 37, 38). Several studies had emphasized that the anti-PD-1 treatment was also capable of promoting vascular normalization, indicating a potential application of CEUS in treatment evaluation among patients treated with anti-PD-1 monotherapy (22, 39). A series of quantitative parameters including TtoPK, PI, grad, and AUC can be acquired from TIC analysis based on CEUS imaging data. These parameters can comprehensively reflect the characteristics of microvessel by depicting the perfusion information and thereby indicate the infiltration status of M2-like macrophages in tumor.

In total, tumor embolus and grad ratio were included as variables in developing our nomogram model. Tumor embolus, presented as enhanced solid areas within the portal vein and its branches in the arterial phase of contrast-enhanced CT/MR images, is a widely used poor prognostic factor for HCC (40–42). In our model, the absence of portal vein embolus was an indicator for non-PD response, which is consistent with a previous study (17). Grad represents the gradient from arrival intensity to PI, reflecting the mean perfusion velocity in concerned regions. Grad depicts the blood flow per unit time and indirectly reflects the microvessel intensity of tumor. Considering that the value of quantitative parameters can be affected by the liver background or the image depth, we introduced the concept of ratio to provide more objective comparison among enrolled patients. The final variable for developing a nomogram was defined as the ratio of the grad derived from TR to that derived from PTR. There was a study addressing that the peritumoral hyper-enhanced ring on the Kupffer phase images obtained *via* Sonazoid-CEUS is a promising marker for predicting the response of anti-PD-1/PD-L1 monotherapy. However, the Sone Vue used in our study was capable of remaining inside the vasculature and avoiding the possibility of being taken by Kupffer cells. The parameters obtained from PTR merely represent the perfusion information of microvessels. According to the odds ratio calculated by logistic regression analysis, patients with lower grad ratios were more likely to benefit from the combined treatment. Considering the positive correlation between microvessel intensity and M2-like TAM infiltration, a lower grad ratio may indirectly reflect the low microvessel intensity in the tumor area, leading to a lower M2-like TAM infiltration and a better treatment efficacy. A nomogram is a practical tool to quantify variables and incorporate multiple variables to establish a prediction model. In our study, ROC curve analysis, calibration curve analysis, and DCA were performed to evaluate the performance of the nomogram and achieved a satisfactory result.

Although a dose gradient of IBI305 (7.5 mg/kg vs. 15mg/kg) was present in our study, there is no significant difference in terms of the IBI305 dose between the PD group and the non-PD group in baseline analysis, excluding the possibility that the difference in IBI305 dose may affect the treatment efficacy. In addition, all enrolled patients received the same therapeutic combination of sintilimab and IBI305 in our study, ensuring the consistency of treatment strategy in each patient. According to RECIST, a patient may be misclassified as non-responder because the tumor size may remain unchanged or slightly increase due to hemorrhage, necrosis, or edema. In order to better evaluate the viable tumor portions, mRECIST was applied to evaluate the response on CT images.

There are several limitations to our study. First, the sample size was relatively small and the survival data like OS and PFS were not included in this study. Second, only internal validation was performed due to the restriction of sample size. Third, patient body habitus, bowel gas, and lesion size and location within the liver may limit imaging access and affect the target lesion selection in some patients. Fourth, operator dependence in the acquisition of sonographic images may limit the generalized application of this prediction model. Therefore, a quantitative CEUS-based prospective study with a larger sample size and detailed survival data was needed to screen the advanced HCC population who may benefit from a combined strategy.

## Conclusions

This study has established and validated a nomogram by incorporating pretreatment CEUS quantitative parameters and baseline clinical characteristics to predict the anti-PD-1 plus anti-VEGF treatment efficacy in advanced HCC patients, which may help in clinical decision-making for patients with advanced HCC.

## Data availability statement

The raw data supporting the conclusions of this article will be made available by the authors, without undue reservation.

## Ethics statement

The studies involving human participants were reviewed and approved by Ethics committee of the cancer hospital of the Chinese Academy of Medical Sciences. The patients/participants provided their written informed consent to participate in this study.

## Author contributions

LN, CS were responsible for the study design and instruction. CS was in charge of the data acquisition, data processing, statistical analysis and manuscript writing. QW was in charge of data preservation and data collection. LH was in charge of data processing and mapping. YC was in charge of paper polishing and revision. All authors contributed to the article and approved the submitted version.
